# Retraction: Lycorine displays potent antitumor efficacy in colon carcinoma by targeting STAT3

**DOI:** 10.3389/fphar.2025.1596508

**Published:** 2025-03-25

**Authors:** Frontiers Editorial Office

The journal retracts the August 8 2018 article cited above.

Following publication, concerns were raised regarding the integrity of the images in the published figures. The authors failed to provide a satisfactory explanation during the investigation, which was conducted in accordance with Frontiers’ policies. As a result, the data and conclusions of the article have been deemed unreliable, and the article has been retracted.

The authors did not provide a response to this retraction.

